# Does glucose-dependent insulinotropic polypeptide receptor blockade as well as agonism have a role to play in management of obesity and diabetes?

**DOI:** 10.1530/JOE-23-0339

**Published:** 2024-07-15

**Authors:** Ryan A Lafferty, Peter R Flatt, Victor A Gault, Nigel Irwin

**Affiliations:** 1 Diabetes Research Centre, Schools of Biomedical Sciences and Pharmacy & Pharmaceutical Sciences, Ulster University, Coleraine, Northern Ireland, UK

**Keywords:** diabetes, glucose-dependent insulinotropic polypeptide, glucagon-like peptide-1, obesity, polypharmacy, satiety

## Abstract

Recent approval of the dual glucose-dependent insulinotropic polypeptide (GIP) and glucagon-like peptide-1 (GLP-1) receptor agonist, tirzepatide, for the management of type 2 diabetes mellitus (T2DM) has reinvigorated interest in exploitation of GIP receptor (GIPR) pathways as a means of metabolic disease management. However, debate has long surrounded the use of the GIPR as a therapeutic target and whether agonism or antagonism is of most benefit in management of obesity/diabetes. This controversy appears to be partly resolved by the success of tirzepatide. However, emerging studies indicate that prolonged GIPR agonism may desensitise the GIPR to essentially induce receptor antagonism, with this phenomenon suggested to be more pronounced in the human than rodent setting. Thus, deliberation continues to rage in relation to benefits of GIPR agonism vs antagonism. That said, as with GIPR agonism, it is clear that the metabolic advantages of sustained GIPR antagonism in obesity and obesity-driven forms of diabetes can be enhanced by concurrent GLP-1 receptor (GLP-1R) activation. This narrative review discusses various approaches of pharmacological GIPR antagonism including small molecule, peptide, monoclonal antibody and peptide-antibody conjugates, indicating stage of development and significance to the field. Taken together, there is little doubt that interesting times lie ahead for GIPR agonism and antagonism, either alone or when combined with GLP-1R agonists, as a therapeutic intervention for the management of obesity and associated metabolic disease.

## Introduction

Glucose-dependent insulinotropic polypeptide (GIP) is a 42-amino acid polypeptide hormone secreted from intestinal K-cells of the duodenum and proximal jejunum (Buchan *et al.* 1978). GIP was discovered in 1969, through collaboration between John Brown and Raymond Pederson at University of British Colombia, Vancouver, alongside Viktor Mutt and Erik Jorpes from Karolinska Institutet, Stockholm (Brown *et al.* 1969, 1970, 1982). Thus, GIP was recognised by endocrinologists over a decade prior to another closely related, but now more widely renowned gut-derived hormone, glucagon-like peptide-1 (GLP-1) (Müller *et al.* 2019). Like GLP-1, GIP is released into the circulation in response to ingestion of macronutrients, it is degraded by dipeptidyl peptidase-4 (DPP-4) and accounts for a major part of the ‘incretin-effect’ by enhancing glucose-stimulated insulin secretion (GSIS) (Pederson *et al.* 1975). GIP exerts its effects on GSIS via binding at GIP receptors (GIPR) on beta cells and activation of cyclic adenosine monophosphate (cAMP) plus associated signal transduction pathways (Ding & Gromada 1997). Additionally, GIP and its receptor are evidenced within the brain, with GIPR expression in the hypothalamus being implicated in the modulation of food intake and satiety, particularly in the arcuate, paraventricular, and dorsomedial nuclei regions (Adriaenssens *et al.* 2019, Samms *et al.* 2020). In addition, GIPR signalling is also demonstrated within circumventricular organs (CVOs), including the area postrema and nucleus tractus solitarius of the dorsal vagal complex (DVC) (Adriaenssens *et al.* 2019, 2023), which are not enclosed by the blood–brain barrier (BBB). Importantly, despite complex interplay between neuronal circuitry within these brain regions, distinct outcomes following GIPR agonism have been confirmed. For example, while hypothalamic GIPRs supress food intake, it appears GIPRs in the DVC may be implicated in taste avoidance (Adriaenssens *et al.* 2023). Use of fluorescently tagged GLP-1 mimetics indicate that exogenous peptides are not thought to cross the BBB but can interact with CVOs (Secher *et al.* 2014), with the same likely to be true for GIP-based compounds. Moreover, GIP possesses peripheral actions to improve insulin action and modulates lipid metabolism that influences overall energy balance whilst also potentially reducing energy intake (Samms *et al.* 2020).

**Figure 1:**
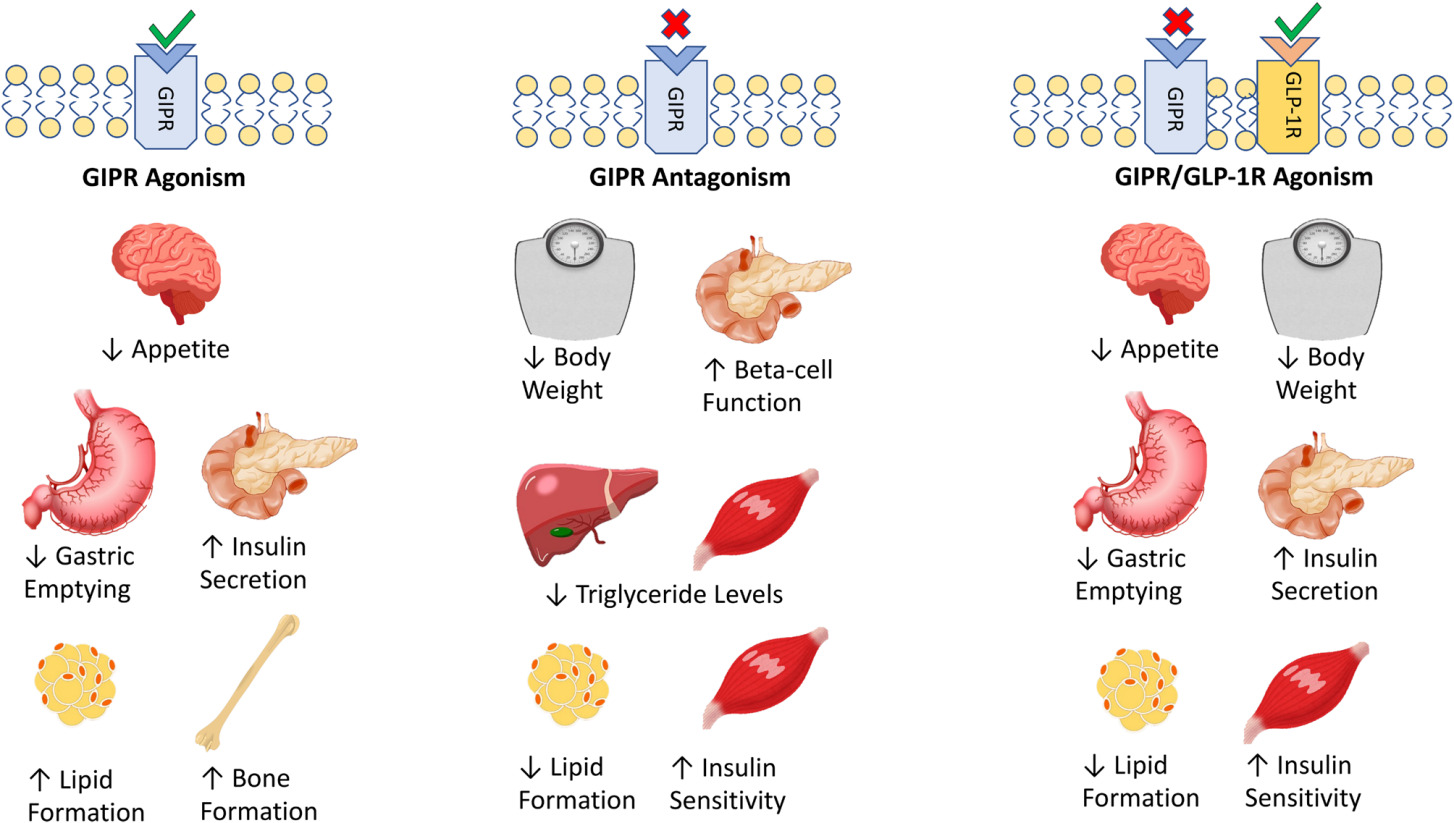
A summary of the tissue-specific benefits of GIPR agonism and antagonism. In addition, the impact of combined GIPR antagonism combined with GLP-1R agonism is considered. Agonism is indicated by green ticks at the GPCR while antagonism is indicated by red crosses. Increases in the therapeutic effect in each instance are indicated by upward arrows and decreases and indicated by downward arrows.

Given the aforementioned biological consequences of GIPR modulation (Fig. 1), it is clear that this signalling pathway holds theoretical promise for the treatment of both type 2 diabetes mellitus (T2DM) and obesity, as has been witnessed to profound effect with GLP-1 (Nauck *et al.* 2021*a*). This is especially relevant since GIP appears to be quantitatively the most important incretin hormone in both rodents and humans (Gault *et al.* 2003*a*, Holst 2019). When we consider that GIP was discovered over a decade prior to GLP-1, it begs the question: Why has the success of GLP-1R mimetics not been emulated or even preceded by GIPR mimetics? The rise of GIP from enterogastrone to major metabolic hormone makes an interesting story (Marks 2020), but the answer is largely due to the well-known insensitivity of humans with obesity and obesity-driven T2DM to the insulinotropic and glucose-lowering actions of GIP (Nauck *et al.* 2021*b*). Such insensitivity can be reversed by lowering blood glucose using insulin, sulphonylureas or DPP-4 inhibitors (Højberg *et al.* 2009, Irwin *et al.* 2010, Stensen *et al.* 2022). However, the situation has not been helped by the debate spanning several decades on whether GIPR agonism or antagonism is most beneficial (Irwin & Flatt 2009*a*, Campbell *et al.* 2022). This review will focus on current evidence that supports the therapeutic promise of GIP and GIPR antagonism and the various approaches taken to impart this.

## GIPR antagonism

The above-mentioned actions of GIP relate primarily to receptor agonism, with positive effects on insulin secretion (Ding & Gromada 1997) and satiety (Samms *et al.* 2020, Fig. 1) having clear potential benefits in obesity and diabetes (Flatt 2008, Irwin & Flatt 2009*b*). However, it is well established that circulating levels of GIP are elevated in human obesity and obesity-driven forms of T2DM (Ebert & Creutzfeldt 1980, Salera *et al.* 1982), with studies using obese rodents reporting expansion of intestinal K-cell mass and elevated circulating GIP in genetically inherited obesity (Flatt *et al.* 1983) and following prolonged exposure to a high-fat, high-calorie diet (Bailey *et al.* 1986).

As such, it has been demonstrated that GIPR-null mice are protected against high-fat-fed-induced obesity and insulin resistance (Miyawaki *et al.* 2002), indicating a role for the GIPR in the onset of obesity (Flatt 2008). In addition, specific destruction of GIP-secreting K-cells in mice also safeguards against diet-induced obesity and ameliorates insulin resistance (Althage *et al.* 2008). Furthermore, various methods to specifically inhibit GIP secretion in rodents are demonstrated to alleviate obesity and insulin resistance (Nasteska *et al.* 2014, Kanemaru *et al.* 2020, Murata *et al.* 2021). Moreover, it is understood that fat is a powerful stimulus for the secretion of GIP which acts at adipocytes. *In vitro* and *ex vivo* studies indicate this increases fat storage (Fig. 1) via upregulation of lipoprotein lipase (LPL) activity (Kim *et al.* 2007) and is associated with phosphorylation of cAMP-response element binding protein (CREB) and nuclear localisation of cAMP-responsive CREB coactivator 2 (TORC2) in human adipocytes (Kim *et al.* 2010). GIPR agonism promotes fatty acid uptake (Killion *et al.* 2020*a*), insulin-induced free fatty acid incorporation into adipocytes (Møller *et al.* 2016) and inhibits lipolysis (Getty-Kaushik *et al.* 2006), whilst improving blood flow to the adipose tissue (Asmar *et al.* 2019). GIPR agonism is also directly implicated in adipocyte growth, with studies in cultured human omental preadipocytes highlighting proliferative actions alongside a reduction in pro-apoptotic transcription factors such as Bcl-2-associated death promoter (BAD) (Chen *et al.* 2021). Importantly, a GIPR antagonist was reported to annul these preadipocyte proliferative effects (Chen *et al.* 2021). However, full* in vivo* characterisation of the mechanisms involved is currently lacking within the literature.

Accordingly, in humans, polymorphisms of the GIPR that lead to perturbed activity are linked to reduced body mass index (Lyssenko *et al.* 2011, Kizilkaya *et al.* 2021). Notably, alterations in G protein coupling and subsequent intracellular signalling cascades with several of these GIPR variants directly mirror consequences of GIPR antagonism (Kizilkaya *et al.* 2021). Additionally, GIP is implicated in increasing cytokine penetration into adipocytes to drive insulin-resistance within these peripheral tissues (Timper *et al.* 2013). Whether this effect is direct or indirect has been debated recently (Campbell *et al.* 2022), but either way it is clear that GIP exerts important effects on adipocyte biology (English *et al.* 2020). In this regard, the literature, based on numerous independent and diverse observations, highlights a clear role for GIPR activation in the development of obesity, grounding the concept of GIPR antagonism as a potential approach to alleviate insulin resistance and excessive weight gain (Irwin *et al.* 2020). While no such therapy has yet made it to clinic, a number of approaches have been employed to impart GIPR blockade including small molecule, immune-neutralisation and peptidic that will be discussed herein.

## Small molecule GIPR antagonism

When considering the extensive body of work linked to the discovery and development of small-molecular weight GLP-1R modulators, including recent work with danuglipron in phase 2 clinical trials (Saxena *et al.* 2023), it is perhaps surprising that a similar literature search in relation to the GIPR heralds much fewer results. Small molecules remain a mainstay of drug development owing to excellent oral bioavailability and reduced production costs when compared to biologics (Beck *et al.* 2022). The desire to generate medications suitable for oral administration is likely the largest driver here, as evident with the retrofitting of Novo Nordisk’s GLP-1R agonist semaglutide, co-formulated with sodium N-(8-(2-hydroxybenzoyl) amino caprylate (SNAC), to prevent destruction of the peptide within the stomach and promote gastric absorption (Bucheit *et al.* 2020). While this formulation of semaglutide, marketed as Rybelsus^®^, represents a significant success in generation of an orally available direct GLP-1R modulator, it is important to note that much greater quantities of peptide are required for oral delivery compared to injectable formulations (14 mg daily vs. 2.4 mg weekly, as respective maximal dosages), which is likely influencing global shortages of the peptide (Whitley *et al.* 2023).

Thus, the appetite for small molecule incretin modulators remains high within the pharmaceutical industry. In the case of GIPR antagonists, only one such agent is described in the literature, termed SKL-14959 (Nakamura *et al.* 2012, Table 1), while no similar GIPR agonist small molecule can be sourced in the literature at the time of writing. SKL-14959 is a potent GIPR antagonist with a molecular weight of less than 400 Daltons and an IC_50_, in relation to cAMP downregulation of 2.9 uM (Nakamura *et al.* 2012). Although SKL-14959 may not be entirely selective, with activity at GLP-1R and the glucagon receptor indicated at concentrations above 3100 and 1000 nM, respectively (Nakamura *et al.* 2012). When evaluated in the acute setting in normal mice, SKL-14959 increased circulating triglyceride levels and reduced LPL and hepatic lipase (HPL) activity following an oil tolerance test (Nakamura *et al.* 2012), which would be indicative of reduced lipid uptake and storage. Additionally, SKL-14959 also effectively countered the actions of exogenously delivered GIP in terms of reducing GSIS during a glucose tolerance test (Nakamura *et al.* 2012).

**Table 1:** A summary of GIPR antagonists with compound classifications, structures, stage of development and supporting references where available. Amino acid sequences are provided for peptide-based antagonists using single-letter abbreviations. ‘Pal’ indicates a C-16 palmitic acid attachment, ‘γE-C16’ indicates a palmitic acid attached via gamma-glutamic acid, ‘Aib’ indicates inclusion of the unnatural amino acid, 2-aminoisobutyric acid. ‘mGIPAb’ is a murine monoclonal antibody while ‘hGIPR’ is human based. Cases in which structures are not available are denoted ‘N/A’.

Compound name	Compound classification	Structure	Stage of development	Company/institution	Reference
SKL-1459	Small molecule	N/A	Preclinical	Sanwa Kagaku Kenkyusho Co.	Nakamura *et al.* (2012, 2018)
GIP(3–42)	Peptide	EGTFISDYSIAMDKIHQQDFVNWLLAQKGKKNDWKHNITQ	Preclinical	University of Copenhagen	Deacon *et al.* (2006), Hansen *et al.* (2016)
GIP(3–30)NH_2_	Peptide	EGTFISDYSIAMDKIHQQDFVNWLLAQK-NH_2_	Preclinical with acute study in humans	University of Copenhagen	Deacon *et al.* (2006), Hansen *et al.* (2016), Sparre-Ulrich *et al.* (2017), Gasbjerg *et al.* (2018)
GIP(7–30)	Peptide	EGTFISDYSIAMDKIHQQDFVNWLLAQK	Preclinical	Boston University	Tseng *et al.* (1999)
GIP(4–42)	Peptide	GTFISDYSIAMDKIHQQDFVNWLLAQKGKKNDWKHNITQ	Preclinical	Ulster University	Kerr *et al.* (2011)
GIP(5–42)	Peptide	TFISDYSIAMDKIHQQDFVNWLLAQKGKKNDWKHNITQ	Preclinical	Ulster University	Kerr *et al.* (2011)
GIP(6–42)	Peptide	FISDYSIAMDKIHQQDFVNWLLAQKGKKNDWKHNITQ	Preclinical	Ulster University	Kerr *et al.* (2011)
GIP(7–42)	Peptide	ISDYSIAMDKIHQQDFVNWLLAQKGKKNDWKHNITQ	Preclinical	Ulster University	Kerr *et al.* 2011
GIP(8–42)	Peptide	SDYSIAMDKIHQQDFVNWLLAQKGKKNDWKHNITQ	Preclinical	Ulster University	Kerr *et al.* (2011)
(Pro^3^)GIP	Peptide	YAPGTFISDYSIAMDKIHQQDFVNWLLAQKGKKNDWKHNITQ	Preclinical	Ulster University	Gault *et al.* (2002)
(Ala^3^)GIP	Peptide	YAAGTFISDYSIAMDKIHQQDFVNWLLAQKGKKNDWKHNITQ	Preclinical	Ulster University	Gault *et al.* (2007*a,b, c*)
(Phe^3^)GIP	Peptide	YAFGTFISDYSIAMDKIHQQDFVNWLLAQKGKKNDWKHNITQ	Preclinical	Ulster University	Gault *et al.* (2007*a*,*b, c*)
(Tyr^3^)GIP	Peptide	YAYGTFISDYSIAMDKIHQQDFVNWLLAQKGKKNDWKHNITQ	Preclinical	Ulster University	Gault *et al.* (2007*a*,* b, c *)
(Hyp^3^)GIP	Peptide	YAOGTFISDYSIAMDKIHQQDFVNWLLAQKGKKNDWKHNITQ	Preclinical	Ulster University	O’Harte *et al.* (2006)
(Hyp^3^)GIP(K^16^Pal)	Peptide	YAOGTFISDYSIAMDK-[Pal]-IHQQDFVNWLLAQKGKKNDWKHNITQ	Preclinical	Ulster University	O’Harte *et al.* (2006)
GIP(3–30)-Cex-K^40^Pal	Peptide	EGTFISDYSIAMDKIHQQDFVNWLLAQKPSSGAPPPSK[Pal]	Preclinical	Ulster University	Pathak *et al.* (2015*a*)
GIP(6–30)-Cex-K^40^Pal	Peptide	EGTFISDYSIAMDKIHQQDFVNWLLAQKPSSGAPPPSK[Pal]	Preclinical	Ulster University	Pathak *et al.* (2015*b)*
Pro^3^GIP(3–30)-Cex-K^40^Pal	Peptide	PGTFISDYSIAMDKIHQQDFVNWLLAQKPSSGAPPPSK[Pal]	Preclinical	Ulster University	Pathak *et al.* (2015*a*)
[N^α^-Ac,L^14^,R^18^,E^21^] hGIP_(5–31)_-K^11^(γE-C16)	Peptide	TFISDYK-[γE-C16]-IALDKIRQQEFVNWLLAQKG	Preclinical	Novo Nordisk	Yang *et al.* (2022)
N^α^Ac,K^10^[γEγE-C16], Arg^18^,hGIP(5–42)	Peptide	TFISDKSIALDK-[γE-C16]-IRQQEFVNWLLAQKGKKNDWKHNITQ	Preclinical	Novo Nordisk	Yang *et al.* (2022)
GIPg013	Monoclonal antibody	N/A	Preclinical	MedImmune / AstraZeneca	Ravn *et al.* (2013)
GIPmAb	Monoclonal antibody	N/A	Preclinical	Case Western Reserve University	Boylan *et al.* (2015)
hGIPR-Ab	Monoclonal antibody	N/A	Preclinical	Amgen	Killion *et al.* (2018)
mGIPR-Ab/P1	Peptide-Monoclonal antibody conjugate	[mGIPAb]-GGGGG-H-Aib-EGTFTSDVSSYLE-Aib-QAAKEFIAWLVKGGG	Preclinical	Amgen	Lu *et al.* (2021)
hGIPR-Ab/P1	Peptide-Monoclonal antibody conjugate	[hGIPAb]-GGGGG-H-Aib-EGTFTSDVSSYLE-Aib-QAAKEFIAWLVKGGG	Preclinical	Amgen	Lu *et al.* (2021)
AMG133	Peptide-Monoclonal antibody conjugate	N/A	Phase 2 to be completed 2025	Amgen	Clinical Trial Identifier: NCT05669599

When assessed in the chronic setting over a 96-day dosing period in high-fat-diet-induced obese (DIO) mice, daily SKL-14959 administration reduced body mass by approximately 7%, an effect that appeared to be independent of food intake (Nakamura *et al.* 2018). Lack of SKL-14959 induced effects on feeding may potentially highlight inability of the molecule to penetrate the BBB and appetite controlling regions within the hypothalamus. In support of observations in the acute setting (Nakamura *et al.* 2012), triglyceride levels in liver, muscle and gastrointestinal muscle were reduced (Nakamura *et al.* 2018), although as the authors concede there is no report of GIPR expression in liver or muscle tissue in rodents (Usdin *et al.* 1993), indicating a likely indirect effect. That said, LPL activity was also reduced that would be linked to a reduction in tissue lipid uptake.

It is unclear why further study of SKL-14959 has not been pursued, especially given promising weight reducing effects in DIO rodents. The compound, which is of unknown structure, appears to be more tolerable than previous attempts of small molecule development against related G protein-coupled receptor (GPCR) targets such as glucagon, which despite clear benefits on diabetes in clinical trials (Cheng *et al.* 2020) were ultimately shelved due to hepatic impairment (Lafferty *et al.* 2022). However, it is noteworthy that despite almost identical binding affinities of SKL-14959 and (Pro^3^)GIP for the GIPR, a peptidic GIPR modulator (Gault *et al.* 2002) did not impart as significant a hyperglycaemic effect when administered daily to normal mice over a period of 11 days (Irwin *et al.* 2004), which is certainly more attractive when considering the target population for a GIPR antagonist treatment. This may have been an important consideration in the halting of development of SKL-14959. Moreover, (Pro^3^)GIP exerted antihyperglycaemic actions in genetically obese diabetic (*ob/ob*) as well as DIO mice (Gault *et al.* 2005, 2007*b*, Irwin *et al.* 2007*a*, McClean *et al.* 2007). That said, there are recent notable species-specific effects of GIPR modulating peptides that also need to be considered when interpreting effects of (Pro^3^)GIP (Sparre-Ulrich *et al.* 2016, Fig. 2), which will be considered in more detail next. Interestingly, 4-hydroxybenzoic acid 2-bromobenzylidene hydrazide (4H2BH) is a small-molecular-weight compound that has been reported to inhibit both GIPR and glucagon receptor activity (Franklin *et al.* 2011), with obvious dual benefits for obesity-related diabetes. However, 4H2BH has not been progressed beyond experiments assessing the impact a single injection in rodents, perhaps suggesting issues with pharmacokinetics and/or safety of this compound.

**Figure 2:**
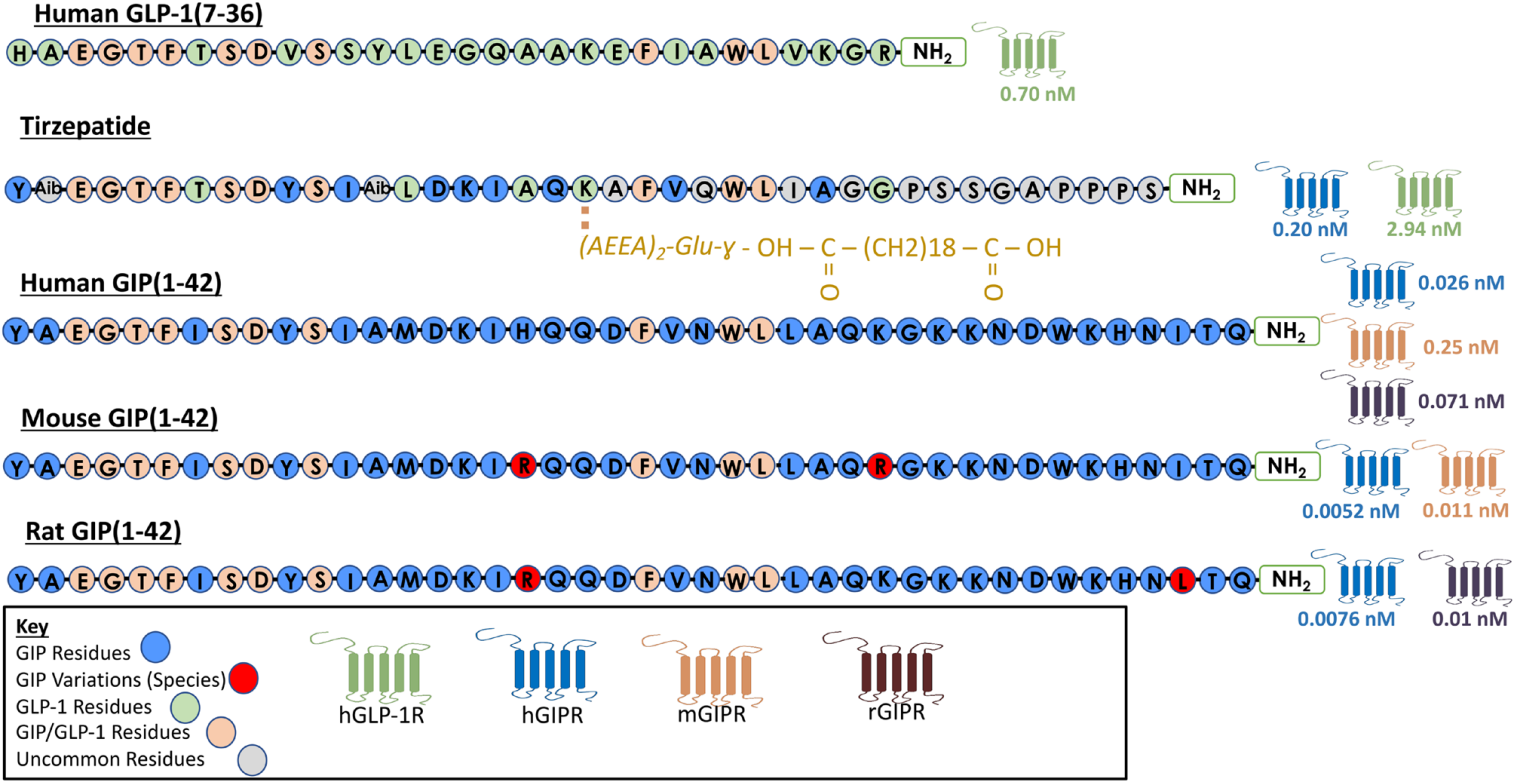
A peptidic structure analysis of glucagon-like peptide-1 (GLP-1) (7–36), the dual GIP/GLP-1 receptor co-agonist tirzepatide and glucose-dependent insulinotropic peptide (GIP) (1–42). Structures for human, mouse and rat GIP(1-42) are provided. Amino acid residues are indicated by single-letter abbreviations. Residues shared with GIP(1–42) are shaded in blue, shared with GLP-1(7–36) are shaded in green, residues shared with both GLP-1 and GIP are indicated in orange and those unique to tirzepatide are shaded in grey. Additionally, species variations between GIP(1-42) are indicated in red. A 20-carbon fatty acid modification, namely eicosanedioic acid, is linked to Glu_20_ with the full structure provided in gold lettering. ‘Aib’ residues indicate inclusion of 2-aminoisobutyric acid, a non-naturally occurring amino acid. Potency at human GLP-1 and GIP receptors (hGLP-1R and hGIPR, respectively) as well as at mouse and rat GIP receptors (mGIPR and rGIPR) are provided, where appropriate, based on EC50 values provided within the literature for each peptide (Sparre-Ulrich *et al.* 2016, Willard *et al.* 2020).

## GIPR immune-neutralisation

A number of studies have indicated that immunisation against GIP peptides, as a means to stimulate active endogenous antibody generation, is an efficacious method of improving obesity-related diabetes in rodents (Fulurija *et al.* 2008, Irwin *et al.* 2009*a*,*b*, 2012, Montgomery *et al.* 2010, Wolfe *et al.* 2023), although suitability of this approach in humans is yet to be determined. Indeed, given the various important physiological actions of GIP (Fig. 1), it may be anticipated that side effects could occur with this approach. Thus, more recent developments have employed administration of GIP monoclonal antibodies as a less permanent method of GIPR blockade, which should decrease side effects risk.

Passive immunity against GIP via administration of monoclonal antibodies (MABs) targeting endogenous GIP, or the GIPR, have proven effective in various studies, either alone or when combined with GLP-1R agonism. While GIP antibodies such as GIPg013 have been developed primarily as a research tool to assess the biological roles and actions of native GIP (Ravn *et al.* 2013, Table 1), others have been investigated as potential pharmacological interventions in obesity. For example, when a GIP MAB, namely GIP mAb (Table 1), targeting the last 17 residues of the C-terminus of murine GIP, was injected once weekly for 17 weeks in normal mice prior to exposure to a high-fat diet, these mice had a remarkable 47% weight loss when compared to untreated control animals (Boylan *et al.* 2015). This was associated with reductions in subcutaneous, abdominal and hepatic fat, with obvious improvements in overall metabolism (Boylan *et al.* 2015). Moreover, these initial findings have been endorsed using human GIPR MABs (hGIPR-Ab; Table 1) in non-human primates (NHP), indicating real potential for translation to the human setting (Killion *et al.* 2018). However, antibody monotherapy in NHPs elicited only a very modest weight reduction of 2%, but when combined with the GLP-1R mimetic dulaglutide, a 15% body weight reduction was observed, which was significantly beyond the 9% reduction achieved with dulaglutide monotherapy (Killion *et al.* 2018).

The apparent synergy between GLP-1R mimetics and GIPR antagonists has been exploited elsewhere in the pursuit of GIP MABs for obesity management (Fig. 1). An exciting new direction is the conjugation of GIPR MABs to peptidic GLP-1R agonists to create a unimolecular dual-acting compound. As such, a recent study by Lu and colleagues reports the successful development of murine and human- based GIPR MABs conjugated to GLP-1(7–37) analogues via a flexible (GGGGS)_3_ linker (Lu *et al.* 2021, Table 1). Administration of the murine-based compound, mGIPR-Ab/P1, over 18 days in DIO mice elicited a 29% reduction in body weight which was associated with reductions in hyperinsulinaemia and cholesterol (Lu *et al.* 2021). Moreover, when the antibody component was delivered alone, a body weight reduction of only 1% was observed, corroborating previous findings of Killion and colleagues on the synergy of this combination therapy approach (Killion *et al.* 2018). Encouragingly, the humanised version of this molecule, hGIPR-Ab/P1, elicited a 14% body weight reduction following 6 weeks administration in obese NHPs although alterations in insulin and cholesterol were less evident than in the corresponding rodent study (Lu *et al.* 2021, Table 1). Mechanistic *in vitro* studies with hGIPR-Ab/P1 indicate a 100-fold increase in cAMP generation when exposed to cells expressing both the GIPR and GLP-1R, when compared to cells expressing one or the other receptor, manifesting in upregulated insulin secretion from INS1 832/3 beta cells (Lu *et al.* 2021). It is thought that dimerisation of the GIPR and GLP-1R in tissues that co-express these receptors allows hGIPR-Ab/P1 to bind simultaneously to both targets and elicit a heightened effect (Whitaker *et al.* 2012, Lu *et al.* 2021). While Lu and colleagues have evidenced this in pancreatic tissues, where GIPR and GLP1-R expression is known to be abundant (Irwin & Flatt 2009*a*), further work is required to confirm the phenomenon in other metabolically relevant sites such as the hypothalamus.

The excellent transition of this dual GIP MAB and GLP-1R agonist approach to NHPs validates appraising this paradigm in the human setting (Fig. 1). Recently, Amgen have completed a phase 1 trial of a GLP-1R mimetic–GIPR antibody conjugate molecule, termed AMG133, which elicited a 15% reduction in body weight over 85 days in its cohort of participants with obesity when administered at the highest dose of 420 mg monthly (Véniant *et al.* 2024, clinical trial identifier: NCT04478708). Excitingly, for subjects receiving either of the highest doses of AMG133 (280 or 420 mg, respectively), 10% body weight was maintained after 150 days of withdrawal (Véniant *et al.* 2024). However, given the lack of comparison against tirzepatide and given the relatively small cohort size, excitement will have to remain tempered for now. A phase 2 study investigating the compound in obese individuals with or without T2DM is currently underway and the results are awaited with great anticipation, estimated to be published in early 2025 (clinical trial identifier: NCT05669599), with hope that answers around the potential for more sustained weight loss with AMG133 can be answered through this trial.

## Peptide-derived GIPR antagonism

Although notable success to annul GIPR signalling has been made with small molecule GIPR modulators and immune neutralisation, this would appear to be more achievable and probably safer through utilising peptide-based ligands of the endogenous receptor. Thus, peptide screening processes such as alanine scanning, or *in silico* molecular conformational software, allows determination of important amino acid residues for peptide activity as well as receptor recognition and binding (Lee *et al.* 2019). In the case of GIP, modification to central amino acid residues is better tolerated for maintaining GIPR agonistic properties than those at the N-terminus (Alaña *et al.* 2006, Venneti *et al.* 2011, Table 1; Fig. 2), with the N-terminus being fundamental for GIPR agonist activity (Hinke *et al.* 2001, Kerr *et al.* 2011, Hansen *et al.* 2016). It is perhaps not surprising that the naturally occurring DPP-4 cleavage products of full length and truncated GIP, namely GIP(3–42) and GIP(3–30) respectively, are antagonists of the GIPR when employed at supraphysiological concentrations (Parker *et al.* 2006, Hansen *et al.* 2016, Table 1). However, at normal circulating concentrations, neither metabolite is thought to have an appreciable impact upon GIPR function and overall metabolism (Deacon *et al.* 2006). Interestingly, further N-terminally truncated GIP metabolites have also been established to possess GIPR antagonistic properties, including GIP(4–42), GIP(5–42), GIP(6–42), GIP(7–42) and GIP(8–42) (Kerr *et al.* 2011, Table 1).

The C-terminally truncated GIP(1–30), found in intestinal K-cells (Fujita *et al.* 2010), has been shown to have similar potency as GIP(1–42) in acute and longer-term studies (Fujita *et al.* 2010, Gault *et al.* 2011). The fragment form, GIP(7–30)NH_2_, was the earliest GIPR antagonist used and effectively demonstrated the importance of GIPR signalling in the insulin response to oral glucose in rats (Tseng *et al.* 1999). However, of the various truncated metabolites, GIP(3–30) is believed to be a highly effective naturally occurring GIPR antagonist (Hansen *et al.* 2016), being superior to GIP(3–42) in terms of inhibiting GIP-induced insulin, glucagon and somatostatin release *in vitro* and in the perfused rat pancreas (Sparre-Ulrich *et al.* 2017). Indeed, GIP(3–30)NH_2_ was the first GIPR antagonist to be utilised in human studies and shown to reduce the GSIS effects of GIP by 82% in healthy volunteers (Gasbjerg *et al.* 2018). Interestingly, no influence on circulating lipid levels was observed following acute GIP(3–30)NH_2_ infusion, but this may be linked to use of a single infusion and the fact that volunteers were healthy (Gasbjerg *et al.* 2018).

Interestingly, whilst the aforementioned peptidic GIPR antagonists have either N- or C-terminal truncation of the GIP amino acid sequence, or a combination of both, an analogue of GIP(1–42), namely DPP-4 resistant (Pro^3^)GIP, appeared to break this mould (Gault *et al.* 2002, Table 1). (Pro^3^)GIP effectively antagonised cAMP-stimulatory action of GIP *in vitro* with an IC_50_ of 2.6 µM whilst also impeding GIP-induced insulin secretion during a glucose tolerance test (GTT) in *ob/ob* mice (Gault *et al.* 2002). In addition, related Glu^3^-substituted analogues of GIP(1–42) were also shown to possess postulated GIPR inhibitory actions (Table 1; O’Harte *et al.* 2006, Gault *et al.* 2007*a*). More intensive study of (Pro^3^)GIP followed, which demonstrated that non-fasting glucose and plasma insulin levels as well as GSIS were not impacted following 11-days once-daily administration of the peptide in non-diabetic mice, thereby suggesting possible compensation by endogenous GLP-1 (Irwin *et al.* 2004). Indeed, GIPR knock-out mice exhibit increased islet sensitivity to GLP-1 (Pamir *et al.* 2003), with once daily injection of (Pro^3^)GIP for 50 days in DIO mice increasing circulating total GLP-1 concentrations (McClean *et al.* 2007). Additional investigations with (Pro^3^)GIP revealed prominent amelioration of insulin resistance and substantial improvements of overall metabolism in *ob/ob* (Gault *et al.* 2005, Irwin *et al.* 2007*a*) as well as DIO mice (Gault *et al.* 2007*b*). Interestingly, benefits were largely absent in streptozotocin (STZ)-treated insulin-deficient mice (McClean *et al.* 2008*a*), suggesting positive effects to be insulin dependent. Encouraging effects on metabolism were also observed when (Pro^3^)GIP was combined with other therapies, such as PYY(3–36) (Irwin *et al.* 2007*b*), cannabinoid CB1 receptor antagonism (Irwin *et al.* 2008), and cholecystokinin (CCK) receptor activation (Irwin *et al.* 2013) as well as GLP-1R agonism (Irwin *et al.* 2009*b*), in keeping with observations of the marked benefits of AMG133 noted previously. Although (Pro^3^)GIP analogues with a protracted duration of biological action have also been characterised (Gault et al 2007*a*, McClean *et al.* 2008*b*), subsequent study revealed differences in the affinity of (Pro^3^)GIP for human and rodent GIPRs and the occurrence of noteworthy species-specific effects of GIP peptides as described further next.

### Species specificity of GIP peptides

There are small, but seemingly important, differences in the sequence of human and rodent GIP, with the human GIP sequence specifically containing His^18^, Lys^30^ and Ile^40^ amongst its 42 amino acid residues, which are substituted with Arg^18^ and Arg^30^ in mouse GIP and then Arg^18^ and Leu^40^ in rat GIP (Bailey 2020, Fig. 2). In agreement, a recent report further highlights physiologically important species- and population-specific evolutionary conservation of the GIP peptide amino acid sequence (Lindquist *et al.* 2022, Fig. 2), although there is less certainty around importance of conservation of the GIPR sequence (Irwin 2020). Indeed (Pro^3^)GIP, that is based on the human GIP amino acid sequence, was shown to display greater affinity for human than mouse or rat GIPRs in transfected cell lines (Sparre-Ulrich *et al.* 2016, Fig. 2), being considered as a low potency GIPR agonist as opposed to a full antagonist (Sparre-Ulrich *et al.* 2016). This suggests that diminished GIP action rather than total GIPR blockade may be sufficient to impart the positive effects in obesity (Gault *et al.* 2005, 2007*b*, Irwin *et al.* 2007*a*, McClean *et al.* 2007). Indeed, this fits well with the clear benefits of GIP immune-neutralisation described earlier, that is unlikely to induce total blockade of GIP action. Interspecies variations of the human, rat and mouse GIP(3–30) sequences have been additionally confirmed through their GIPR antagonist capabilities, with each peptide recognised as a true competitive GIPR antagonist only within their respective parent systems (Gabe *et al.* 2018, Perry *et al.* 2019). Furthermore, whilst the GIPg013 GIPR antibody reported earlier effectively antagonised mouse, rat dog, and human GIPRs, a closely related GIPR antibody, Gipg133, had no GIPR antagonist activity at mouse and rat GIPRs (Ravn *et al.* 2013).

Despite concerns over species variation (Fig. 2), human (Pro^3^)GIP(3–30)-based peptides have since been described that possess full GIPR antagonist activity in rodent systems (Pathak *et al.* 2005, Table 1). This discovery shadowed initial findings with a GIP(3–30) based-peptide, namely GIP(3–30)-Cex-K^40^PAL, that combines GIP(3–30) with the nine C-terminal residues of the GLP-1R agonist, exendin(1–39) (Pathak *et al.* 2015*a*, Table 1), where this C-terminal extension, Cex, was previously demonstrated to improve metabolic stability and reduce renal clearance of GLP-1 peptides (Simonsen *et al.* 2013). An additional C-terminal lysine residue was also attached to the molecule at position 40, K^40^PAL, to facilitate attachment of a C-16 fatty acid that prolongs the bioactivity profile (Pathak *et al.* 2015*a*, Table 1). Modification to the C-terminus of GIP has previously been reported to interfere less with ligand-receptor binding (Hinke *et al.* 2001). As an additional means to ensure adequate receptor engagement, the molecule was also N-terminally capped with phenyl lactic acid to preserve the helical structure (Doig & Baldwin 1995, Pathak *et al.* 2015*a*). Importantly, GIP(3–30)-Cex-K^40^PAL and the related Pro^3^GIP(3–30)-Cex-K^40^PAL molecules were determined to effectively antagonise the actions of native GIP with nanomolar potency, specifically in terms of cAMP recruitment in human GIPR-transfected Chinese Hamster Lung cells in addition to a reduction of GIP-induced insulin secretion from rodent BRIN-BD11 cells (Pathak *et al.* 2015*a*). More significantly, the GIPR antagonist peptides were then assessed in DIO mice and shown to induce sustained weight loss, counter insulin resistance and improve glycaemic control following once daily injection for 21 days (Pathak *et al.* 2015*a*). Indeed, Pro^3^GIP(3–30)-Cex-K^40^PAL was the better performing of the two peptides and body weight at study termination in this group of mice was not significantly different from non-obese controls. It is also interesting to note that these mice presented with a reduction of fat mass, but no obvious impact on lean mass, indicating an appropriate manner of weight loss (Pathak *et al.* 2015*a*), that may not be the case with some GLP-1 mimetics (Lafferty *et al.* 2023). Generally speaking, the phenotype induced by GIP(3–30)-Cex-K^40^PAL and Pro^3^GIP(3–30)-Cex-K^40^PAL were similar to those observed previously with Pro^3^GIP (McClean *et al.* 2007).

Finally, a related GIPR antagonist peptide, namely GIP(6–30)Cex-K^40^PAL, exhibited profound benefits on metabolism in diabetic *db/db* mice, but particularly so when added to liraglutide treatment (Pathak *et al.* 2015*b*), further supporting the notion of the significant therapeutic promise of combined GIPR antagonism and GLP-1 agonism (Fig. 1). Interestingly, there is a school of thought that GIPR antagonism can impart beta-cell resting benefits (Gault *et al.* 2005, Tanday *et al.* 2022), to help protect chronically over-activated beta cells and prevent their apoptosis (Fig. 1). In this respect, it is notable that GIP(6–30)Cex-K^40^PAL and liraglutide were administered sequentially in *db/db* mice by Pathak and colleagues to impart scheduled periods of beta-cell rest and activation (Pathak *et al.* 2015*b*), which may represent a treatment paradigm worthy of further consideration.

More recently another acylated GIPR antagonist peptide has been reported in the literature, termed (N^α^-Ac, L^14^, R^18^, E^21^) hGIP(5–31)-K^11^(γE-C16) (Yang *et al.* 2022, Table 1). This GIP(5–31) analogue seems to have directly arisen from an earlier reported C-terminally intact GIP analogue, namely N^α^Ac, K^10^(γEγE-C16), R^18^, hGIP(5–42), which was studied in a head-to-head comparison with GIPR agonist peptides (Mroz *et al.* 2019, Table 1). (N^α^-Ac, L^14^, R^18^, E^21^) hGIP(5–31)-K^11^(γE-C16) was shown to exert modest reductions of food intake and body weight following 27 days administration in DIO mice (Yang *et al.* 2022). More interestingly, when combined with the GLP-1R agonist, semaglutide, these mice displayed increased appetite suppression and body weight loss as well as a modest improvement of glucose tolerance when compared to semaglutide monotherapy (Yang *et al.* 2022), in good support of previous observations utilising this GLP-1R agonism and GIPR antagonism treatment paradigm (Pathak *et al.* 2015*b*; Fig. 1). To date, none of these longer acting, acylated GIPR antagonist peptides have been evaluated in humans, but the use of suitably characterised humanised versions may represent the next major advancement in this area of research.

## Does prolonged GIPR agonism equate to antagonism?

Considerable excitement has surrounded the emergence and clinical approval of tirzepatide, the first-in-class GIPR/GLP-1R co-agonist peptide. Tirzepatide was shown to elicit up to 10% body weight reduction and substantial decrease in waist circumference following 12 weeks administration in T2DM patients during phase 2 trials (Frías *et al.* 2021), manifesting in an average weight loss of 11.2 kg in the group receiving the highest dose of tirzepatide compared to an average weight loss of 5.7 kg in participants receiving semaglutide. There may be a further benefit in this combination owing to a proposed anti-emetic effect of GIPR activation at the area postrema (Borner *et al.* 2021), which is the vomiting centre of the brain and could alleviate nauseating side effects of GLP-1R agonists.

Despite these marked benefits, the precise mechanism of action of tirzepatide remains unclear. The peptide amino acid sequence bears a striking resemblance to human GIP, in keeping with the strong preference of tirzepatide towards the GIPR over the GLP-1R (Willard *et al.* 2020, Fig. 2). Fascinatingly, there is a growing body of evidence suggesting that prolonged GIPR activation desensitises the GIPR *in vitro*, essentially then mimicking GIPR antagonism (Campbell 2021, Gasbjerg *et al.* 2023*b*). This desensitisation is postulated to be the result of reduced GIPR recycling to the cell-membrane surface following initial activation and internalisation, as evidenced in 3T3-L1 adipocytes (Mohammad *et al.* 2014), as well as primary rodent adipose tissue exposed to a long-acting GIPR agonist for 24 h (Killion *et al.* 2020*a*). While *ex vivo* confirmation of GIPR desensitisation following prolonged GIPR agonism in adipocytes indicates relevance of this phenomenon *in vivo* (Killion *et al.* 2020*a*), comparable data in other tissues is currently lacking. Moreover, prolonged GIPR agonism exerted clear benefits on bone strength and composition in insulin-resistant high-fat-fed mice (Vyavahare *et al.* 2020), as well as improving cognition (Siano *et al.* 2011), suggesting lack of receptor desensitisation in these tissues. This is strengthened by studies in type 2 diabetic patients, where GIPR agonism clearly reduces bone resorption (Christensen *et al.* 2020) despite postulated beta-cell insensitivity to GIP in this population (Nauck *et al.* 1993). However, the desensitisation perspective is supported by a study comparing administration of a long-acting GIPR agonist and a GIPR mAb in DIO mice, where both approaches elicited almost identical reductions in body weight when employed as monotherapy (< 5%) or when combined with liraglutide (~20%) (Killion *et al.* 2020*a*). Moreover, recent evidence appears to indicate that the human GIPR may be more prone to desensitisation through internalisation than the murine GIPR (Gasbjerg et al 2023*a*), that may be an important consideration when evaluating the translational applicability of GIPR antagonists.

Further to this, biased agonism at the level of GLP-1R has also been suggested with tirzepatide (Xiao et al 2023) that leads to favoured cAMP generation over β-arrestin recruitment for GLP-1R but not GIPR, activation (Gasbjerg et al 2023*b*). However, this needs to be considered in the context that tirzepatide binds with greater preference to GIP rather than GLP-1 receptors (Willard *et al.* 2020). GIPR desensitisation also appears to hold more weight to its argument than the theory of incretin receptor compensation, which surmises that when either incretin receptor is knocked out, sensitivity for the opposite hormone is improved (Campbell 2021), as is its therapeutic effect. However, double incretin-receptor knock-out mice also display reduced weight gain when exposed to a high-fat diet (Hansotia *et al.* 2007). Thus, while a compensatory phenomenon may play a partial role when antagonising a singular receptor, it is unlikely to be the primary mechanism at play when considering the therapeutic benefits GIPR antagonism in combination with GLP-1R agonism.

## Further research and mode of action of tirzepatide

The remarkable story of tirzepatide has substantially reinvigorated interest in the GIPR as a drug target. However, more research is needed to determine the molecular action of this dual GIP/GLP-1 analogue and to assess whether GIPR agonism in this context equates to antagonism. Our laboratory at Ulster has been a strong advocate for the exploitation of GIP therapeutics since the late 1990s, providing substantial evidence for beneficial metabolic effects of both agonism and antagonism of the GIPR (Gault *et al.* 2003*b*,*c*, Irwin & Flatt 2009*a*). This position appeared counterintuitive to many (Meier & Nauck 2004, Seino *et al.* 2010), but may now have greater appeal, given that the action of tirzepatide is believed to possibly involve desensitisation of the GIPR (Gasbjerg *et al.* 2023*a*). Thus, as evident from our early preclinical studies (Gault *et al.* 2005, McClean *et al.* 2007), diminished action or antagonism of GIP substantially decreases obesity-driven insulin resistance by depleting liver triglycerides, reducing adiposity, and thereby substantially diminishing insulin demand with the induction of beneficial beta-cell rest and decreased circulating insulin. As noted above, subsequent studies using our next generation of GIP antagonist peptides, namely GIP(3–30)-Cex-K^40^PAL, Pro^3^GIP(3–30)-Cex-K^40^PAL or GIP(6–30)Cex-K^40^PAL, evoked remarkably similar effects (Pathak *et al.* 2015*a*,*b*). This scenario clearly contrasts with the more obvious and predominantly insulin-releasing incretin effects triggered by GIP agonism, which would predominate if tirzepatide acted simply as a dual GIP/GLP-1 agonist. Another mechanistic pathway postulated recently by Gasbjerg and colleagues is that tirzepatide acts solely as a GLP-1R super agonist (Gasbjerg *et al.* 2023*b*), but this possibility seems less appealing based on the more impressive effects of tirzepatide over those of GLP-1 mimetics in both humans and animal models with obesity-diabetes (Coskun *et al.* 2018, Frias *et al.* 2020*b*). Additionally, the aforementioned binding preference of tirzepatide towards the GIPR, and high sequence homology with native GIP, would tend to cast aspersions on this argument (Willard *et al.* 2020, Fig. 2). Future acute studies in man looking at the efficacy of tirzepatide alone and in combination with the GIPR antagonist GIP(3–30) or the GLP-1R antagonist exendin(9–39) can be expected to help to resolve this issue and shape the development of future GIP-incorporating compounds.

## Conclusion

The resounding therapeutic success of the GIPR/GLP-1R co-agonist, tirzepatide, has seen a resurgence of interest in GIPR modulation for the management of T2DM and obesity (Nauck & Müller 2023). While it appears that the pendulum has begun to swing in favour of GIPR agonism over GIPR antagonism for imparting metabolic benefits, significant debate remains given recent evidence that prolonged GIPR activation may lead to GIPR desensitisation (Killion *et al.* 2020*a*,*b*), particularly in the human setting (Gasbjerg et al 2023*a*). This becomes even more relevant given the receptor preference of tirzepatide towards the GIPR (Willard *et al.* 2020). In addition to this, it is apparent that metabolic advantages of GIPR antagonism can be enhanced through concomitant GLP-1R activation (Fig. 1), as evidenced by preclinical studies (Gault *et al.* 2005, 2007*a*, *b*, 2011, Irwin *et al.* 2009*a*,*b*, Pathak *et al.* 2015*a*,*b*) and progression of the conjugated MAB GIPR antagonist/GLP-1R agonist therapy, AMG133 (Véniant *et al.* 2024), to phase 2 clinical trials. Finally, the organ-specific effects of GIPR agonism and antagonism must be ascertained, particularly in relation to desensitisation given the wealth of evidence supporting a central mechanism for GIPR-agonist-induced reductions in food intake (Seino *et al.* 1997, Adriaenssens *et al.* 2019, Samms *et al.* 2020), but with no current evidence supporting a role for desensitisation of GIPR in appetite-regulating centres of the brain through prolonged exposure. Thus, with continuing development on GIP/GLP-1R co-agonists modalities (Lafferty *et al.* 2023), it is hoped that clarity can be ascertained as to whether GIPR agonism or antagonism has the greatest role to play in management of obesity and related metabolic diseases.

## Declaration of interests

PRF, VAG and NI are named on patents filed by Ulster University for the exploitation of incretin-based drugs and other peptide therapeutics. RAL, VAG, PRF and NI are shareholders in Dia Beta Labs Ltd., an Ulster University spinout developing peptide therapeutics for the management of metabolic disease.

## Funding

This work did not receive any specific grant from any funding agency in the public, commercial, or not-for-profit sector.
